# Maternal Passive Smoking, Vitamin D Deficiency and Risk of Spontaneous Abortion

**DOI:** 10.3390/nu14183674

**Published:** 2022-09-06

**Authors:** Shiqi Lin, Jiajia Li, Yuan Zhang, Xinming Song, Gong Chen, Lijun Pei

**Affiliations:** 1 Institute of Population Research and China Center on Population Health and Development, Peking University, Beijing 100871, China; 2 National Research Institute for Health and Family Planning, Beijing 100081, China

**Keywords:** passive smoking, vitamin D deficiency, spontaneous abortion, co-exposure

## Abstract

Background: Maternal passive smoking and vitamin D deficiency might elevate risk of spontaneous abortion. The study aimed to investigate the association of co-exposure to passive smoking and vitamin D deficiency with the risk of spontaneous abortion. Methods: A population-based case-control study was performed among non-smoking women in Henan Province, China, with 293 spontaneous abortion cases and 496 liveborn controls with term, normal birthweight. Results: Compared to women without exposure to passive smoking nor vitamin D deficiency, women with deficient vitamin D alone and women with exposure to passive smoking alone had increased risk of spontaneous abortion (OR = 1.76, 95%CI: 1.08~2.89; OR = 1.73, 95%CI: 1.11~2.69, respectively). The risk of spontaneous abortion was even higher for those with co-exposure to passive smoking and vitamin D deficiency (OR = 2.50, 95%CI: 1.63~3.84). A dose-response relationship was found of an incremental risk of spontaneous abortion with rising numbers of exposures to passive smoking and vitamin D deficiency (*p* < 0.001). Conclusion: Co-exposure to passive smoking and vitamin D deficiency was associated with an elevated risk of spontaneous abortion, and the risk of spontaneous abortion rose with rising numbers of exposures. Intervention programs need to specifically target the vulnerable groups of pregnant women with both malnutrition and unfavorable environmental exposure.

## 1. Introduction

Spontaneous abortion is often accompanied by early and late maternal complications including blood loss, infection and symptomatic complaints, such as pain and bleeding, together with objective difficulties in conceiving [1]. It is also often a sentinel predictor of several adverse outcomes in subsequent pregnancies such as neural defects, recurrent spontaneous abortion or perinatal mortality [2,3]. In China, it is reported that the incidence of spontaneous abortion is around 10~14% [4,5]. Besides genetic and demographic factors, spontaneous abortion can also be attributed to acquired and environmental factors, many of which are modifiable [2].

Cigarettes contain hundreds of toxic substances such as nicotine, cotinine, carbon monoxide, volatile organic compounds or polycyclic aromatic hydrocarbons (PAH), lead and cadmium. With high lipid solubility, a number of these substances could rapidly cross the placenta, accumulate and metabolize in the fetus, causing up to twice the concentration of cotinine on the fetal than on the maternal side [6] and threatening the developing fetus [7]. Currently, evidence from human observational studies of the association between spontaneous abortion and active smoking is more conclusive, yet studies concerning passive smoking are fewer, results are inconsistent, and most were performed in Western countries [8]. In China, the prevalence of passive smoking for non-smoking women was considerable, reaching around 40% in the workplace, 51% in the home and even 76% in restaurants [9,10]. Women in rural areas or those who are less educated tend to be more easily exposed to passive smoking [11,12,13]. A large population-based retrospective cohort study in China indicated that women exposed to their husbands’ smoking during preconception had an 11% (95% CI: 1.08~1.14) increased risk of spontaneous abortion compared with those without such exposure [14]. Another case-control study also found a rising risk of unexplained recurrent spontaneous abortion among women self-reported to be exposed to passive smoking compared to the non-exposed group [15]. However, more evidence is needed concerning the potential effects and mechanisms of passive smoking on the risk of spontaneous abortion in the Chinese population.

Among pregnant women in China, vitamin D deficiency is prevalent, with an insufficiency prevalence rate of 45% and a deficiency rate of 42% [16]. Circulating vitamin D appears even lower in smokers [17], probably because of tobacco’s role in disrupting the vitamin D endocrine system [18] and causing low intake of vitamin D through changing dietary taste. Moreover, maternal vitamin D deficiency might endanger the maintenance of pregnancy and, thus, lead to spontaneous abortion, a conjecture that has been supported by some epidemiological studies [19,20]. Hence, it is likely that co-exposure to passive smoking and vitamin D deficiency corresponds to a higher risk of spontaneous abortion than a single exposure. However, past literature has scarcely probed into this topic. Therefore, based on data from rural Henan Province, China, the present study aimed to explore the relationship among passive smoking, vitamin D deficiency and risk of spontaneous abortion.

## 2. Materials and Methods

### 2.1. Study Design and Data Collection

A population-based case-control study was derived from the Birth Defects Monitoring and Comprehensive Intervention Project in Henan Province, China from December 2009 to January 2010. As the fifth most populous province [21], Henan is in central China with latitude/longitude of 31°23′ N–36°22′ N/110°21′ E–116°39′ E. Detailed study design has already been described elsewhere [20,22]. In brief, in order to obtain a representative sample of women of child-bearing age, we conducted a multi-stage cluster sampling of women between 18 and 40 years old with permanent local registered residency. A total of 1151 participants had their serum blood collected, among whom 293 had a spontaneous abortion within one year and 498 had term (≥37 gestational weeks) normal birthweight (≥2500 g and <4000 g) liveborn babies without birth defects during the same period [20]. As our study focused on the effects of passive smoking, we excluded women with active smoking (*n* = 2). Finally, there were 789 participants included in our study, with 293 spontaneous abortion cases and 496 controls.

After recruitment, participants were interviewed face-to-face by trained healthcare workers about their basic socio-demographic information, as well as their behavioral and dietary habits during pregnancy. During the interview, 8 mL of women’s fasting venous blood was also collected, prepared by centrifugation and then stored at −80 °C at Peking University until analysis.

The study protocol was reviewed and approved by the Institutional Review Board of Peking University Health Science Center. All subjects gave written informed consent before completing the questionnaire and collection of blood samples.

### 2.2. Spontaneous Abortion Cases and Controls

Spontaneous abortion cases in our study were defined as clinically recognized pregnancy loss before 28 gestational weeks. Controls were women delivering liveborn babies with neither birth defects nor preterm birth (<37 gestational weeks), low birthweight (<2500 g) or high birthweight (≥4000 g).

### 2.3. Maternal Passive Smoking and Vitamin D Deficiency

Maternal passive smoking was defined according to the survey question, “Did you passively inhale cigarette smoke by smokers around you for an average of more than 15 min per day during pregnancy?”. Vitamin D was measured quantitatively based on the level of serum 25-hydroxyvitamin D (25(OH)D) concentration in serum samples, which were analyzed using the high-performance liquid chromatography-tandem mass spectrometry (HPLC-MS/MS, Ultimate3000–API 3200 Q TRAP) method. Here we defined vitamin D deficiency as 25(OH)D < 20 ng/mL and sufficiency as ≥20 ng/mL as suggested by previous literature [23].

### 2.4. Covariates

Women’s basic characteristics, including age, history of chronic diseases, education, occupation, and diet and behavioral factors, were considered as potential covariates in the analysis. An extreme BMI could have a negative role in reproductive outcomes as well as vitamin D status [2,24] and, thus, was also evaluated as a potential covariate. BMI was calculated as weight divided by the square of height. We used 24 kg/m^2^ and 28 kg/m^2^ as cut-off points for the “normal weight or underweight”, “overweight” and “obesity” groups given the specific body characteristics of Chinese population [25]. Diet referred to vitamin D supplementation (supplementation of vitamin D, cod-liver oil or any multivitamins containing vitamin D), nutritional supplementation (any supplementation of vitamin A, multivitamin B, vitamin B1, vitamin B2, vitamin B6, vitamin B12, vitamin C, vitamin E, cod-liver oil or vitamin D, iron preparations, calcium tablets or zinc), and average frequencies of intake of food that contained relatively high vitamin D, namely, meat, aquatic products, eggs, and milk or dairy products. Behavioral habits were alcohol consumption (drinking alcoholic beverages more than once a month) and physical exercises (referring to any indoor or outdoor health-promoting physical exercises more than once a week for more than 30 min per time). As serum 25(OH)D levels might be influenced by sunlight exposure, the sampling time was also considered.

### 2.5. Statistical Analysis

Univariate analysis with $\chi^{2}$ test (or Fisher’s exact test when appropriate) was performed to test the differences of socio-demographic characteristics, dietary and behavioral factors as well as sampling time between spontaneous abortion cases and controls and between those with vitamin D deficiency and sufficiency. Significant variables (*p* < 0.05) were then included in later multivariate models.

We conducted a multivariate logistic regression model to investigate the association of passive smoking and vitamin D deficiency with risk of spontaneous abortion. To explore the single and combined effects of maternal passive smoking and vitamin D deficiency, we first divided participants into four groups: (1) women without passive smoking nor vitamin D deficiency; (2) those without passive smoking but with vitamin D deficiency; (3) those without vitamin D deficiency but with passive smoking and (4) those with passive smoking and vitamin D deficiency. The four groups represented a rising degree of the two exposures. Multivariate models were then performed by comparing the latter three groups with the first group to evaluate risk of spontaneous abortion across different combinations of the two risk factors. A trend test was then conducted to determine whether the risk increased with rising degree of exposure combinations. We also conducted an interaction test [26] to determine whether there was an interaction effect between the two factors.

## 3. Results

The basic characteristics, behavioral and dietary habits and vitamin D status of spontaneous abortion cases and controls were displayed in Table 1. Our study mainly included women ≥ 28 years old (62%), achieving junior high school or lower educational attainment (76%) and being a housewife or farmer (81%). Alcoholic consumption was rare among participants, with only 13 (1.7%) reporting drinking over once per month. While passive smoking was prevalent, with over 60% participants demonstrating that they were exposed to smoking air for an average of over 15 min per day. The serum samples were collected after pregnancy outcomes and all in winter (Jan and Dec). Vitamin D deficiency was common, accounting for 49% of all participants. Significant differences were observed between spontaneous abortion cases and controls with regard to BMI, history of chronic diseases, meat intake, milk intake, passive smoking and vitamin D deficiency (*p* < 0.05).

Supplemental Table S1 displays the differences in basic characteristics between different vitamin D statuses. Table 2 describes the results of multivariate analysis for the association of passive smoking and vitamin D deficiency with risk of spontaneous abortion. The multivariate logistic regression analysis showed that women exposed to passive smoking were associated with a 57% (95%CI: 1.15~2.14) higher risk of spontaneous abortion compared to unexposed women; women with vitamin D deficiency were also associated with a higher risk of spontaneous abortion (OR = 1.56, 95%CI: 1.15~2.10).

Table 3 showed the different risk of spontaneous abortion across different exposure combinations of passive smoking and vitamin D status. Compared to women without passive smoking nor vitamin D deficiency, a 1.73 (95%CI: 1.11~2.69) times higher risk of spontaneous abortion was observed among those without vitamin D deficiency but with passive smoking, and 1.76 (95%CI: 1.08~2.89) times higher risk for those without passive smoking but with vitamin D deficiency, after adjusting for BMI, history of chronic diseases, meat intake and milk intake. The risk of spontaneous abortion was even higher, accounting for 2.50 (95%CI: 1.63~3.84) times for those with both passive smoking and vitamin D deficiency. There was no statistical interactive effect observed (*p* = 0. 82). A significant $\chi_{trend}^{2}$ (*p* < 0.001) indicated a dose-response relationship of an increased risk of spontaneous abortion with rising degree of combined exposure to passive smoking and vitamin D deficiency.

## 4. Discussion

### 4.1. Main Findings

With a population-based case-control study performed in rural Henan Province, China, we explored the association between maternal passive smoking, vitamin D status and risk of spontaneous abortion. The findings indicated a dose-response relationship of incremental risk of spontaneous abortion with rising degree of combined exposure to passive smoking and vitamin D deficiency. Our study provided clues of the combined effects of passive smoking and nutritional deficiency on the risk of adverse pregnancy outcomes in a Chinese population of reproductive age.

Our study found that there was an association between maternal exposure to passive smoking and an elevated risk of spontaneous abortion. This result was consistent with another case-control study in China that showed a rising risk of unexplained recurrent spontaneous abortion for passive smokers [15]. A case-control study in Sweden also indicated a higher probability of having a history of spontaneous abortion in women with exposure to passive smoking (defined as plasma cotinine concentrations from 0.1 to 15.0 ng/mL) [27]. Potential mechanisms could be the hazardous substance in tobacco smoke accumulating in women’s bodies and crossing the placenta to induce maternal complications and placental pathology [28] affecting the fetal development [7] and, thus, predicting spontaneous abortion [29]. Previous research on the relationship between smoking and spontaneous abortion focuses mainly on active smokers [8]. However, albeit active smoking is relatively rare in pregnant women, passive smoking is still severe in China [9]. Provided that non-smokers with passive smoking generally inhale much smaller amounts of tobacco smoke particles and nicotine than an active smoker [30], our results that passive smoking was associated with a 57% increased risk of spontaneous abortion echoes the warning from the WHO that there is no safe level of tobacco smoke exposure [31]. What is more, nonsmokers with passive smoking are influenced by not only mainstream but also sidestream smoke, which contains different quantities of toxic substances from the former. In other words, passive and active smoking might act differently on maternal and fetal health. As it is well-recognized that the origins of spontaneous abortion are multifactorial and it is hard to observe the whole process and find mechanisms, epidemiological studies are important to provide clues, especially for environmental risk factors. Our study, thus, adds evidence from the Chinese population for avoiding maternal exposure to passive smoking during pregnancy to reduce risk of adverse pregnancy outcomes, including spontaneous abortion.

Furthermore, our findings indicated that the risk of spontaneous abortion increased by 137% for those with co-exposure to passive smoking and vitamin D deficiency (OR = 2.37, 95%CI: 1.54~3.67) compared to those with neither exposure. Furthermore, there was a dose-response relationship of increased risk of spontaneous abortion with the incremental numbers of exposures to passive smoking and vitamin D. Our previous research has already found that women with vitamin D deficiency were more likely to have experienced spontaneous abortion [20]. As stated above, passive smoking might also be associated with a higher risk of spontaneous abortion. What is worse, smoking might further lead to vitamin D deficiency [17], probably because smoking brings about disorders of food intake, synthesis, hydroxylation and catabolism of vitamin D [18]. That could explain our finding that a combined exposure of passive smoking and vitamin D deficiency might be related to an even higher risk of spontaneous abortion than single exposure. This co-exposure to malnutrition and unfavorable environment is constantly observed in some socio-economically disadvantaged groups. Explanations have been offered by past researchers that women with low socio-economic status, low educational level or unprofessional jobs are inclined to be exposed to passive smoking and vitamin D deficiency [11,12,13,22,32]. Our previous study showed that lower maternal socio-economic status (SES) was associated with higher risk of vitamin D deficiency in women of childbearing age in rural northern China [22], and low maternal SES may strengthen the effect of vitamin D deficiency exposure on spontaneous abortion risk [20]. The two studies, along with the present one, thus, call for policy makers and the whole society to offer special attention to periconceptional women in disadvantaged socio-economic groups with overlying exposure to undernutrition and harmful environments.

### 4.2. Strengths and Limitations

One of the strengths of the present study was that it is a population-based case-control study adopting a representative and relatively large sample, thus increasing the test power. The selection bias was minimized with a multi-stage cluster sampling method. By restricting our subjects to non-smokers, we excluded the potential confounding effects of active smoking. Furthermore, compared to methods using spousal smoking as a surrogate of exposure to passive smoking, our method, which asked women whether they were surrounded by a smoking environment, was more direct because, with the spread of health knowledge today, many smokers would avoid smoking at home, especially with their pregnant wives or children.

The limitations of our study should also be acknowledged. First, we regarded the measurement of vitamin D level after the pregnancy outcomes as an estimate of vitamin D exposure during pregnancy by assuming a stable serum 25(OH)D level. Admittedly, this was a very strong assumption and serum 25(OH)D does change over time when sunlight, temperature, weight status, nutritional supplementation and other factors change. A longitudinal study that tracked serum 25(OH)D levels found a low degree of agreement in two waves with a 14-year interval [33]. However, we measured the serum 25(OH)D about one year after the pregnancy outcomes, a relatively short period during which serum 25(OH)D level usually had a high consistency according to past research [34,35]. Additionally, vitamin D supplementation was not a prevalent practice among young women in China, as can be indicated in our study and others that only a very small proportion of women took vitamin D supplementation (9% in our study and 9% in another one conducted in 2010–2012 [16]). What is more, the effects of vitamin D deficiency are presumably cumulative instead of temporal. Therefore, our way of estimating cumulative vitamin D exposure during pregnancy was reasonable. Second, the passive smoking was self-reported instead of by some biomarkers that proved to be a more precise way of exposure measurement [36]. However, many past researchers have implied that self-reported questions concerning passive smoking status had a high consistency to nicotine in biomarkers and, thus, could be a reliable way to assess passive smoking in adults [37]. Even if the misclassification was brought about by women’s self-report, it is most probably a nondifferential one [38]. Third, our study did not collect more detailed information about the sources and duration of passive smoking exposure, while the degree of exposure might be different with regard to various conditions. Future studies can consider collecting more information on exposure such as whether the exposure comes from home or the workplace and on average how many cigarettes are smoked around the women.

## 5. Conclusions

In summary, through a population-based case-control study in Henan Province, China, our study investigated the individual and combined effects of maternal passive smoking and vitamin D deficiency during pregnancy on risk of spontaneous abortion. It was found that there was an association of maternal exposure to passive smoking and vitamin D deficiency with an elevated risk of spontaneous abortion. Moreover, compared to women without exposure to passive smoking nor vitamin D deficiency, the risk of spontaneous abortion rose when the exposures increased. Our study speaks to the importance of effective education and public health intervention programs specifically targeting vulnerable groups of pregnant women with both malnutrition and unfavorable environmental exposure.

## Figures and Tables

**Table 1:** Differences in basic characteristics between spontaneous abortion cases and controls.

Variables	Cases (*n* = 293)	Controls (*n* = 496)	*p*
	*n* (%)	*n* (%)	
Basic characteristics			
Age (years)			0.75
<28	110 (37.5)	192 (38.7)	
≥28	183 (62.5)	304 (61.3)	
Education			0.15
High school or above	61 (20.8)	125 (25.3)	
Junior high or below	232 (79.2)	369 (74.7)	
Occupation			0.74
Unemployed or famers	55 (18.8)	98 (19.8)	
Others	238 (81.2)	398 (80.2)	
Household annual income (RMB ^1^)			0.34
≥10,000	172 (58.7)	307 (62.2)	
<10,000	121 (41.3)	187 (37.8)	
BMI (kg/m^2^)			
<24	170 (58.0)	324 (65.3)	0.03
24–28	79 (27.0)	126 (25.4)	
>28	44 (15.0)	46 (9.3)	
History of chronic diseases			
No	243 (82.9)	449 (90.5)	0.002
Yes	50 (17.1)	47 (9.5)	
Dietary habits			
Nutritional supplement			0.36
No	216 (73.7)	380 (76.6)	
Yes	77 (26.3)	116 (23.4)	
Vitamin D supplement ^2^			0.25
No	261 (89.1)	454 (91.5)	
Yes	32 (10.9)	42 (8.5)	
Meat intake			0.02
≥once per week	144 (49.1)	285 (57.6)	
<once per week	149 (50.9)	210 (42.4)	
Aquatic product intake			0.09
≥once per month	51 (17.4)	111 (22.4)	
<once per month	242 (82.6)	384 (77.6)	
Eggs intake			0.15
Everyday	92 (31.4)	157 (31.7)	
4–6 times per week	60 (20.5)	129 (26.1)	
≤3 times per week	141 (48.1)	209 (42.2)	
Milk or dairy products intake			0.006
≥4 times per week	49 (16.7)	100 (20.2)	
<4 times per week but at least once per month	70 (23.9)	159 (32.1)	
Almost never	174 (59.4)	236 (47.7)	
Behavioral factors			
Alcohol consumption			0.92
No	288 (98.3)	488 (98.4)	
Yes	5 (1.7)	8 (1.6)	
Physical exercise			0.93
No	251 (85.7)	422 (85.4)	
Yes	42 (14.3)	72 (14.6)	
Passive smoking			0.004
No	97 (33.1)	215 (43.4)	
Yes	196 (66.9)	281 (56.6)	
Vitamin D status			0.003
Sufficient	129 (44.0)	273 (55.0)	
Deficient	163 (56.0)	223 (45.0)	
Sampling time			0.34
January 2010	42 (14.3)	84 (16.9)	
December 2009	251 (85.7)	412 (83.1)	

^1^ RMB: the Chinese official currency; ^2^ sufficient: 25(OH)D ≥ 20 ng/mL, deficient: 25(OH)D < 20 ng/mL.

**Table 2:** Analysis of association of passive smoking and vitamin D deficiency with risk of spontaneous abortion.

Exposure	cOR(95%CI)	aOR(95%CI) ^1^
Passive smoking	1.55(1.14~2.09)	1.57(1.15~2.14)
Vitamin D deficiency ^2^	1.56(1.16~2.08)	1.56(1.15~2.10)

^1^ Adjusted for BMI, milk or diary product intake, meat intake and history of chronic diseases; ^2^ vitamin D deficiency: 25(OH)D < 20 ng/mL.

**Table 3:** Risk of spontaneous abortion across different combination groups of passive smoking and vitamin D status.

Passive smoking	Vitamin D Deficiency ^1^	Cases (*n* = 293)	Controls (*n* = 497)	aOR (95%CI) ^2^	*p*
No	No	130	46	1.00	
Yes	No	143	83	1.73(1.11~2.69)	<0.05
No	Yes	85	51	1.76(1.08~2.89)	<0.05
Yes	Yes	140	113	2.50(1.63~3.84)	<0.001
$\chi_{trend}^{2}$					<0.001
Interaction					0.82

^1^ Vitamin D deficiency: 25(OH)D < 20 ng/mL; ^2^ adjusted for BMI, milk or diary product intake, meat intake and history of chronic diseases.

## Data Availability

The data are available from the corresponding author upon reasonable request.

## Supplementary Materials

The following supporting information can be downloaded at: https://www.mdpi.com/article/10.3390/nu14183674/s1, Table S1: Differences of basic characteristics between different vitamin D status.
