# Supplementary material for: Maternal Passive Smoking, Vitamin D Deficiency and Risk of Spontaneous Abortion
Source: Nutrients. 2022 Sep 6;14(18):3674. doi: 10.3390/nu14183674 (PMC9501103; doi:10.3390/nu14183674)
Supplement: Supplementary file 1 [file nutrients-14-03674-s001.zip › nutrients-1864849-supplementary.pdf]

Supplemental Table S1. Differences of basic characteristics between different vitamin D status

Variables	Vitamin D deficiency(n=387)	Vitamin D suffciency (n=402)	P
	n(%)	n(%)	
Basic characteristics			
Age			0.003
<28	128(33.07)	174(43.28)	
≥28	259(66.93)	228(56.72)	
Education			0.12
High school or above	82(21.24)	104(25.94)	
Junior high or below	304(78.76)	297(74.06)	
Occupation			0.048
Unemployed or famers	301(77.78)	335(83.33)	
Others	86(22.22)	67(16.67)	
Household annual income (RMB[1])			0.03
≥10,000	219(56.88)	260(64.68)	
< 10,000	166(43.12)	142(35.32)	
BMI (kg/m2)			
＜24	245(63.31)	249(61.94)	0.14
24-28	91(23.51)	114(28.36)	
>28	51(13.58)	39(9.70)	
History of chronic diseases			
No	340(87.86)	352(87.56)	0.90
Yes	47(12.14)	50(12.44)	
Dietary habits			
Nutritional supplement			0.053
No	304(78.55)	292(72.64)	
Yes	83(21.45)	110(27.36)	
Vitamin D supplement[2]			0.08
No	358(92.51)	357(88.81)	
Yes	29(7.49)	45(11.19)	
Meat intake			0.90
≥ once per week	211(54.66)	218(54.23)	
< once per week	175(45.34)	184(45.77)	
Aquatic product intake			0.81
≥ once per month	78(20.21)	84(20.90)	
< once per month	308(79.79)	318(79.10)	
Eggs intake			0.004
Everyday	118(30.57)	131(32.59)	
4-6 times per week	76(19.69)	113(28.11)	
≤ 3 times per week	192(49.74)	158(39.30)	
Milk or dairy products intake			0.37
≥ 4 times per week	76(19.69)	73(18.16)	
<4 times per week but at least once per month	119(30.83)	110(27.36)	
Almost never	191(49.48)	219(54.48)	
Behavioral factors			
Alcohol consumption			0.26
No	383(98.97)	393(97.76)	
Yes	4(1.03)	9(2.24)	

Physical exercise			0.08
No	338(87.79)	335(83.33)	
Yes	47(12.21)	67(16.67)	
Passive smoking			0.01
No	136(35.14)	176(43.78)	
Yes	251(64.86)	226(56.22)	
Sampling time			<0.001
Jan, 2010	82(21.19)	44(10.95)	
Dec, 2009	305(78.81)	358(89.05)	

[1] RMB: the Chinese official currency; [2] Sufficient: 25(OH)D≥20 ng/mL, Deficient: 25(OH)D <20 ng/mL
